# Vaccine Biomarkers: In Search of a Goldilocks Approach

**DOI:** 10.1016/j.ebiom.2018.03.013

**Published:** 2018-03-21

**Authors:** Ali M. Harandi

**Affiliations:** Department of Microbiology & Immunology, Institute of Biomedicine, University of Gothenburg, Medicinaregatan 7A, Gothenburg, Sweden

The Roman encyclopaedist Aulus Cornelius Celsus (c. 25 BC – c. 50 AD) known for his extant medical work “De Medicina” is commemorated for recording the first markers of inflammation known as “Celsus tetrad”. Two millennia later in the 21^st^ century, the underlying molecular and cellular mechanisms of Celsus tetrad signs of inflammation are unravelled, and a few blood biomarkers of inflammation such as TNF-α and IL-1β are used in clinical practice.

Antibodies have long served as the correlate of immunogenicity and efficacy for the majority of human vaccines across different target groups. Recently, systems serology offers an unbiased approach to systematically profile antibody functional response characteristics that may be generated following vaccination. Such systematic serological approaches are aimed at capturing the biodiversity in antibody profiles that may emerge following vaccination. This can presumably identify the features of humoral immunity that distinguish protective from non-protective responses, which can in turn inform a rational development of vaccines. Although antibodies serve as reliable vaccine biomarkers for diseases that are deemed to be prevented by antibody responses, they do not represent a biomarker of choice for vaccines that mediate their protective effect through cell-mediated immunity. Furthermore, the development of antibody responses takes weeks to months following vaccination, and as such antibodies fail to serve as an early blood biomarker of vaccine immunogenicity and efficacy. Such early biomarkers can help with the down selection of top vaccine candidates among existing candidates early in vaccine research and development. This is of paramount importance especially during outbreaks of emerging and re-emerging infections.

Systems vaccinology is an emerging field that employs cutting-edge *-omics* technologies, such as genomics, transcriptomics, proteomics, miRNomics and metabolomics, combined with conventional immunological read-outs and systems biology approaches for a holistic systems analysis of human responses to vaccination. Owing to the hypothesis-generating nature of systems biology approaches, to explore potential mechanisms, molecular signatures identified must to be investigated for cause–effect relationships in animal experimentation, and then validated in nested cohort human trials (a co-clinical approach). Recently, systems analysis of human vaccines has begun to yield new insights into the mechanisms of action of several human vaccines and adjuvants. This movement is also deemed to advance the biomarkers of vaccine immunogenicity and efficacy beyond antibodies by discovery of early blood molecular biomarkers that correlate with, and even predict, the later vaccine-specific adaptive immune responses and protection against diseases. If successful, this can potentially save resources and accelerate vaccine R&D, thus leading to enhanced preparedness in response to emerging and re-emerging outbreaks. However, a few technological hurdles, including a need for harmonization and standardization of *-omics* technology platforms and computational/systems biology approaches, remain to be negotiated to enhance reproducibility of the *-omics* data, and to allow inter-laboratory comparisons. A few central issues need to be addressed before new biomarkers of vaccine immunogenicity and efficacy proposed by systems vaccinology could be translated to the clinic. First, how realistic is it to discover early blood biomarkers specific for each human vaccine to be able to enhance vaccine biomarkers beyond conventional specific antibody and T cells? Similar to other biological systems, the immune system operates in a resource-efficient manner by for example employment of a number of shared protective mechanisms to counter different pathogens or to respond to vaccination. It is therefore plausible that distinct molecular biomarkers of immune responses are discovered for different classes of vaccines, e.g., live attenuated viral vaccines, alum-adjuvanted recombinant protein vaccines and conjugate vaccines, rather than biomarkers excusive to each individual vaccine within each vaccine class. In keeping with this notion, a recent study by Bali Pulendran’s group showed distinct transcriptional signatures of vaccine-specific antibody responses to different modalities of human vaccines. In other words, vaccines with similar composition are likely to share common molecular signatures.

Notwithstanding, it should be noted that a vaccine biomarker may be merely a collateral effect of the vaccine, and may be completely unrelated to protection or the clinical endpoint. Second, it remains to be established whether early biomarkers of vaccines are universal for different target groups, e.g., adults vs the very young and very old, well-nourished vs malnourished people, and people living in the areas where the diseases, for which vaccines are purposed, are endemic. It is proposed that the investment of the body’s limited resources in innate and acquired immunity induced following infection or vaccination is optimized, among other factors, in response to local environmental conditions such as nutritional abundance and the degree of pathogen exposure. Hence, early biomarkers of vaccine-induced responses in healthy adults living in developed countries may not overlap those of infants, elderly or people living in resource-poor countries. Last, systems vaccinology employs cutting edge expensive technologies, and sophisticated bioinformatics and systems biology approaches. Translation of the novel biomarkers of vaccines emerging from *-omics* approaches to the clinic needs the development of simple, robust, and preferably low-cost technologies.

It is envisaged that based on the rapid pace of investigation in the field, integration of data generated from state-of-the-art immunological read outs and cutting-edge *-omics* technologies will yield a more nuanced and holistic understanding of correlates of vaccine immunogenicity, efficacy, and reactogenicity to inform rational design of safe and efficacious vaccine strategies. Nonetheless, research on vaccine biomarkers has so far received little attention as an independent scientific priority from most of the main research-funding agencies and policy makers. More efforts are needed to highlight the importance of vaccine biomarkers on the global vaccine R&D agenda, and to foster collaboration and flow of information among different stakeholders.

## Disclosure

The author declares no conflict of interest.

